# The possibility to make choices modulates feature-based effects of reward

**DOI:** 10.1038/s41598-019-42255-1

**Published:** 2019-04-08

**Authors:** Anna Heuer, Christian Wolf, Alexander C. Schütz, Anna Schubö

**Affiliations:** 1 Experimental and Biological Psychology, Philipps-Universität Marburg, Marburg, Germany; 2 Present Address: Department of Psychology, Humboldt-Universität zu Berlin, Rudower Chaussee 18, 12489 Berlin, Germany

## Abstract

When making decisions, humans can maximize the positive outcome of their actions by choosing the option associated with the highest reward. We have recently shown that choices modulate effects of reward via a bias in spatial attention: Locations associated with a lower reward are anticipatorily suppressed, as indicated by delayed responses to low-reward targets and increased parieto-occipital alpha power. Here, we investigated whether this inhibition also occurs when reward is not coupled to location but to a nonspatial feature (color). We analyzed reaction times to single targets associated with a low or high reward as a function of whether a second trial type, choice-trials, were interleaved. In choice-trials, participants could choose either one of two targets to obtain the associated reward. Indeed, responses to low-reward targets were slower when choice-trials were present, magnifying the influence of reward, and this delay was more pronounced in trials immediately following a choice. No corresponding changes in parieto-occipital alpha power were observed, but the behavioral findings suggest that choices modulate a reward-related bias in feature-based attention in a similar manner as for spatial attention, and support the idea that reward primarily affects behaviour when it is of immediate relevance.

## Introduction

Humans value specific events or objects differently, and strive to optimize their behavior by choosing actions that are associated with a rewarding outcome. Reward is an important incentive that shapes not only big decisions in life, but also our experience of the world around us at a much more fundamental level by modulating the processing of visual information. Reward influences which parts of our visual surroundings we attend to and thus select for further processing^1,2^, where and when we look^3–5^, what we remember^6–8^ and how we perform even simple, visually-guided movements such as reaching^9,10^.

Biasing processing in a reward-related manner is only truly advantageous when the outcome of a given situation or event depends on a person’s behavior. This is for instance the case when choices can be made between multiple options of varying associated value. Indeed, we have recently shown that the necessity or possibility to make choices modulates effects of reward on response preparation of saccadic eye movements^11^ and manual responses^12^. Both studies employed a task that consisted of two trial types, which were presented in an interleaved fashion. In single-trials, participants had to respond to a single target presented to the left or right from fixation. Each target was associated with either a low or a high reward and participants received these rewards for correct responses. In choice-trials, both targets were presented and participants were free to choose to obtain the associated reward. Reward was coupled to the spatial location(s) of the target(s): One visual hemifield was assigned a low reward, and the other one a high reward. We manipulated the proportion of choice-trials within a block of trials, and analyzed single-trial performance as a function of choice-trial proportion in the same block. Across several experiments, response latencies to low-reward single-targets were delayed when choice-trials were present as compared to blocks with only single-trials. The magnitude of this delay increased with choice-trial proportion and was particularly pronounced in single-trials directly following a choice-trial. Cueing single-trials on a trial-to-trial basis reduced but not eliminated latency differences between low- and high-reward targets, suggesting that the expectation of an upcoming choice-trial could not fully account for the delayed responses to low-reward targets. Importantly, this slowing of responses to low-reward targets was not affected by the frequency of left and right responses or by a change in response from one trial to the next^11^. Taken together, these findings indicate that a stronger reward-related bias was implemented when choice-trials were present, preparing for optimal, reward-maximizing decisions.

Analysis of oscillatory brain activity during a preparatory period preceding target presentation revealed that the underlying reward-related bias was not in motor preparation, but in visuospatial attention. In blocks with a high choice-trial proportion, an increase in parieto-occipital alpha power contralateral to the visual hemifield associated with a low reward was observed prior to target presentation^12^. Preparatory modulations of posterior oscillatory power in the alpha band (8–14 Hz) have been identified as a reliable index of the voluntary deployment of covert visuospatial attention, reflecting the facilitation of relevant and the suppression of irrelevant information via a modulation of cortical excitability in a retinotopic fashion^13–19^. Specifically, increases in posterior alpha power have been suggested to reflect the functional inhibition of the sensory brain areas processing information at the to-be-ignored regions of space^20^. Thus, our previous findings^12^ indicate that when the likelihood was high that a choice could be made to maximize reward, the spatial region associated with a low reward was actively suppressed in preparation for optimal target selection, presumably by reducing baseline excitability^21^. Modelling saccade latency distributions provided further support for this conclusion, likewise indicating that the delayed responses to low-reward single targets in the presence of choice-trials were due to a reduced baseline level^11^.

In our previous studies^11,12^, items were associated with different magnitudes of reward based on their spatial location. However, the value of visual objects cannot only be determined by their location, but also by non-spatial characteristics, which can be simple visual features such as color or size, or more complex stimulus attributes such as object category. This might, in fact, be the more common case in everyday life. In the current study, we examined whether the possibility to make choices in order to maximize positive outcome modulates the effects of reward when reward magnitude is coupled to a non-spatial feature in a similar manner as it does when reward is determined by spatial location^11,12^.

In analogy to our findings with respect to effects of spatially defined reward^12^, we assumed that choices would modulate the effects of featurally defined reward via a reward-related bias in feature-based attention. Feature-based attention selectively increases sensitivity to specific features across the visual field and thereby prioritizes the visual processing of behaviorally relevant stimuli^22,23^. Feature-based and spatial attention thus serve the same function (i.e., to tune visual perception to what is important) and they have similar effects on neuronal responses in visual cortex^24,25^, activating a largely overlapping frontoparietal network^26–28^. In spite of these commonalities, however, feature-based and spatial attention appear to be distinct attentional mechanisms that independently enhance relevant visual signals with dissociable behavioral signatures^29–32^, and that are supported by specialized regions within the common network^26–28,33,34^. Thus, what has been established for spatial attention does not necessarily apply to feature-based attention.

To determine whether the possibility to make choices between options of different value modulates feature-based effects of reward as it does for spatially indicated reward, we modified the task used in our previous studies^11,12^ and coupled reward to color. Delayed responses to low-reward single targets in blocks with choice-trials would indicate that choices do not only induce a reward-related bias in visuospatial attention^11,12^ but also in feature-based attention.

Our prior work identified a spatially specific increase in alpha power, anticipatorily inhibiting regions associated with a low reward, as the neural mechanism that mediated the choice-induced modulation of reward effects^12^. Therefore, we additionally analyzed parieto-occipital alpha power during a preparatory period preceding target presentation to examine whether a reward-related bias in feature-based attention is supported by the same oscillatory mechanism. However, whereas the involvement of alpha oscillations in spatial attention has been well established^14,20^, only few studies have investigated whether preparatory feature-based attention is similarly reflected in alpha power changes, and these studies produced somewhat mixed evidence^35–37^. Inducing the expectation of an upcoming target feature (left- or rightward motion) has been found to increase overall alpha power over occipital cortex as compared to when participants had no expectation^35^. A more specific pattern of alpha-band increases has been observed for different feature dimensions, consistent with the hypothesized role of alpha as a suppression mechanism in spatial attention: When participants were cued to attend to either the color or the motion of an upcoming dot array, alpha power in the cortical areas processing the irrelevant feature dimension increased, indicating their functional inhibition^36^. A recent study directly comparing spatial, feature-based and combined spatial and feature-based cues, by contrast, failed to observe any alpha power modulations specifically related to feature-based attention, even though the feature-based cues were behaviorally effective^37^. Thus, we did not expect that a choice-induced modulation of feature-based reward effects, analogous to our previous findings for spatially indicated reward, would necessarily be reflected in a corresponding modulation of parieto-occipital alpha power. But we tentatively hypothesized that an anticipatory suppression of low-value target features might result in an alpha power increase in neural subpopulations coding for the low-value target feature, reflected in overall higher parieto-occipital alpha power in blocks with choice-trials compared to block without choice trials (see also de Lange *et al*.^35^).

## Results

**Figure 1:**
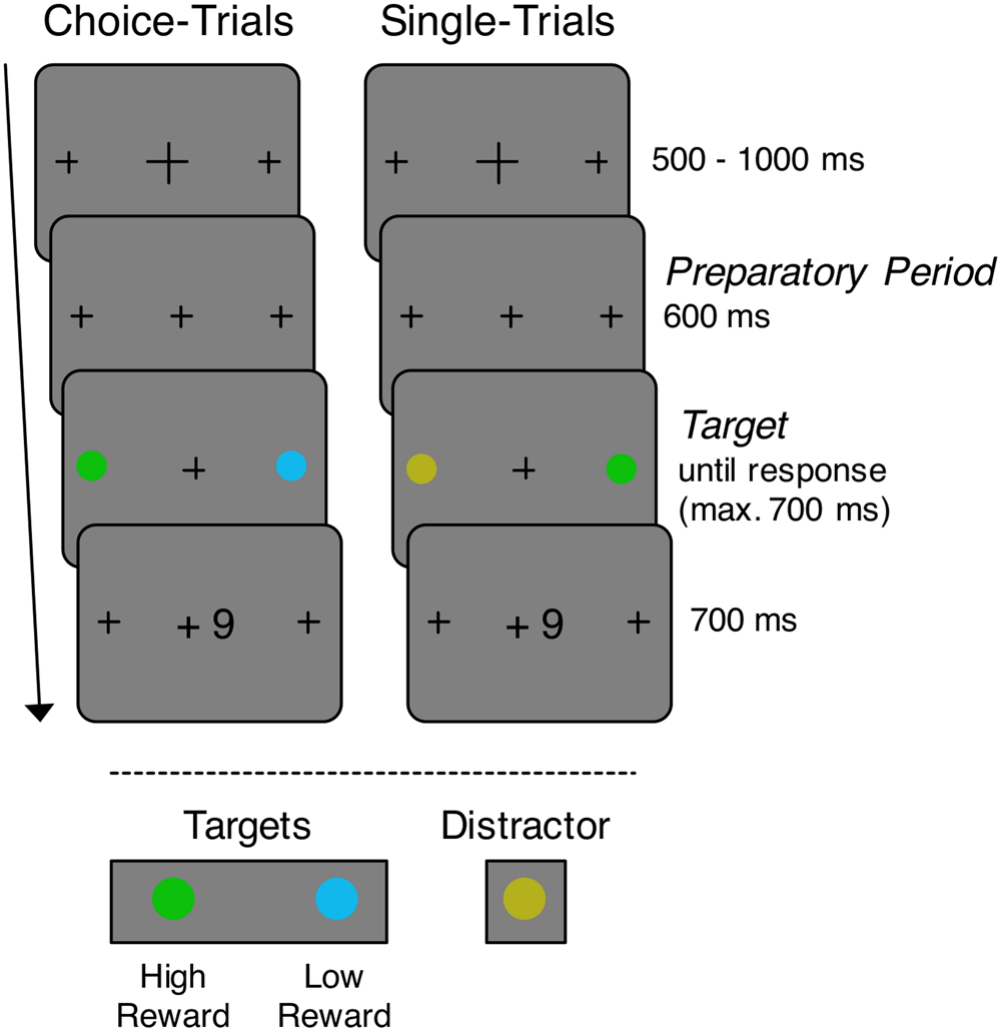
Trial procedure for choice-trials (left) and single-trials (right). A trial started with the presentation of a fixation cross (500–1000 ms, varied in steps of 100 ms). The fixation cross then changed its size to that of the placeholders presented to the left and right, indicating the onset of the target(s) in 600 ms (*preparatory period*). For each participant, two out of three possible colors were defined as target colors. The color assignment was balanced across participants. In the example shown here, green and blue are target colors, and yellow is the distractor color. In single-trials, only one of the circle-shaped items that replaced the placeholders after the preparatory period was of a target color, and participants were to indicate whether that target was presented to the left or right. In choice-trials, both placeholders were replaced by targets, and participants were free to choose either one. The target was present until response or for a maximum of 700 ms. In each block of trials, one target color was associated with a high (9 points) and the other with a low reward (1 point). In single-trials, participants received the corresponding reward for correct responses. In choice-trials, participants received the reward associated with the color of the chosen target.

In this experiment, participants had to indicate the location of a target presented along with a non-rewarding distractor item (single-trials). The task is illustrated in Fig. 1. Targets were defined by their color: Each participant was assigned two target colors, and a third color was used for distractors. One target color was assigned a low reward and the other one a high reward. Participants received these rewards for correct responses. Across blocks of trials, we manipulated the proportion of choice-trials (0 vs. 0.33) that were randomly interleaved with these single-trials. In choice-trials, two targets were presented and participants could choose between the two to obtain the reward associated with the color of the chosen target.

### Behavioral measures

**Figure 2:**
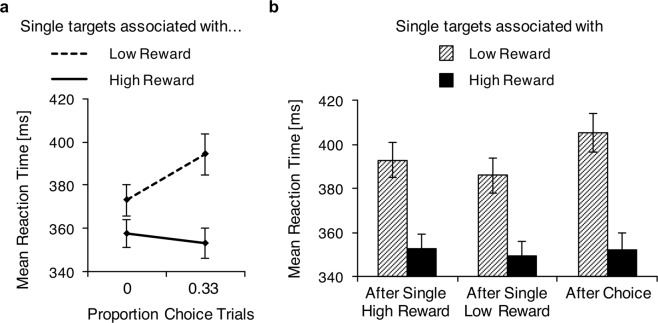
Behavioral results. (**a**) Mean reaction times in single-trials as a function of the proportion of choice-trials (0 to the left, 0.33 to the right) in the same block. The dashed line represents reaction times to targets in the color associated with low reward, the solid line reaction times to targets in the color associated with high reward. Error bars show standard errors of the means. (**b**) Mean reaction times in single-trials as a function of the preceding trial type (after a single trial with a high-reward target, after a single-trial with a low-reward target, and after a choice-trial), shown separately for targets associated with a low reward (striped bars) and for targets associated with a high reward (black bars). Error bars show standard errors of the means.

Our primary measure of interest were single-trial reaction times as a function of choice-trial proportion. In Fig. 2a, their means are shown as a function of the proportion of interleaved choice-trials and separately for low- and high-reward single targets. Responses were slower in the 33% choice-trial condition (373 ms ± 8 ms) than in the 0% choice-trial condition (366 ms ± 7 ms; F_(1,22)_ = 7.40, p = 0.013, partial ƞ^2^ = 0.25), and slower for targets associated with a low reward (384 ms ± 8 ms) than for targets associated with a high reward (356 ms ± 7 ms; F_(1,22)_ = 35.91, p < 0.001, partial ƞ^2^ = 0.62). Importantly, an interaction between choice-trial proportion and reward (F_(1,22)_ = 66.14, p < 0.001, partial ƞ^2^ = 0.75) revealed that the difference in reaction times to low- and high-reward targets was larger in the 33% choice-trial condition (41 ms ± 6 ms) than in the 0% choice-trial condition (15 ms ± 4 ms). Whereas responses to low-reward targets were slower with 33% choice-trials (394 ms ± 9 ms) than without choice-trials (373 ms ± 7 ms; t_(22)_ = 5.43, p < 0.001), responses to high-reward targets were similar in the 0% choice-trial condition (357 ms ± 6 ms) and in the 33% choice-trial condition (353 ms ± 7 ms; t_(22)_ = 1.53, p = 0.141). Thus, the presence of choice-trials slowed responses to low-reward targets, but did not affect responses to high-reward targets.

Single-trial accuracy was close to optimal (94.35% ± 0.73%) and reflected the pattern found for reaction times, ruling out the possible influence of a speed-accuracy trade-off. For accuracies, there was an effect of choice-trial proportion (F_(1,22)_ = 19.25, p < 0.001, partial ƞ^2^ = 0.47) and an interaction between choice-trial proportion and reward (F_(1,22)_ = 16.80, p < 0.001, partial ƞ^2^ = 0.43), but no main effect of reward (F_(1,22)_ = 1.93, p = 0.018, partial ƞ^2^ = 0.08). Whereas accuracy for high-reward targets was at the same level in the 0% (96.06% ± 0.68%) and in the 33% choice-trial condition (96.31% ± 1.13%; t_(22)_ = 0.20, p = 0.84), accuracy for low-reward targets decreased when choices were present (89.32% ± 1.96%) compared to when there were no choices (93.30% ± 1.15%; t_(22)_ = 2.53, p = 0.019). In choice-trials, participants chose the target with high reward in 87.1% of trials, revealing that they indeed aimed to maximize their financial outcome.

We further examined intertrial effects in the 33% choice-trial condition to check whether the delay in responses to low-reward targets was particularly pronounced immediately following a choice-trial^11^. Single-trials in the 33% choice-trial condition were split into trials following either a single-trial with a high-reward target, a single-trial with a low-reward target, or a choice-trial. Figure 2b shows reaction times in single-trials separately for these different preceding trial types and for low- and high-reward targets. Overall, responses were faster for single targets associated with a high reward than for single targets associated with a low reward (F_(1,22)_ = 80.57, p < 0.001, partial ƞ^2^ = 0.79). Reaction times were also found to be influenced by the preceding trial type (F_(2,44)_ = 9.42, p < 0.001, partial ƞ^2^ = 0.30): Responses were fastest after a single-trial with a low-reward target (368 ms ± 7 ms), slightly slower after a single-trial with a high-reward target (373 ms ± 7 ms), and slowest after a choice-trial (379 ms ± 7 ms). Importantly, there was also an interaction (F_(2,44)_ = 4.23, p = 0.021, partial ƞ^2^ = 0.16), indicating that the difference in reaction times to low- and high-reward targets differed depending on the preceding trial. To further elucidate this interaction, we conducted separate one-way ANOVAs for low- and high reward targets. For low-reward targets, there was an effect of preceding trial type (F_(2,44)_ = 10.323, p < 0.001, partial ƞ^2^ = 0.32) and subsequent pairwise comparisons revealed that responses following a choice-trial were slower than responses following a single-trial with a low-reward target (19 ms ± 5 ms, p = 0.002) and responses following a single-trial with a high-reward target (13 ms ± 3 ms; p = 0.003). Responses following the two types of single-trials did not differ significantly (7 ms ± 5 ms, p = 0.456). Reaction times to high-reward targets were not affected by the preceding trial type (F_(2,44)_ = 0.57, p = 0.571, partial ƞ^2^ = 0.03). Thus, the effect of reward on single-trial performance was larger when the previous trial was a choice-trial, and this was due to a slowing of responses to low-reward targets.

### Parieto-occipital alpha power

**Figure 3:**
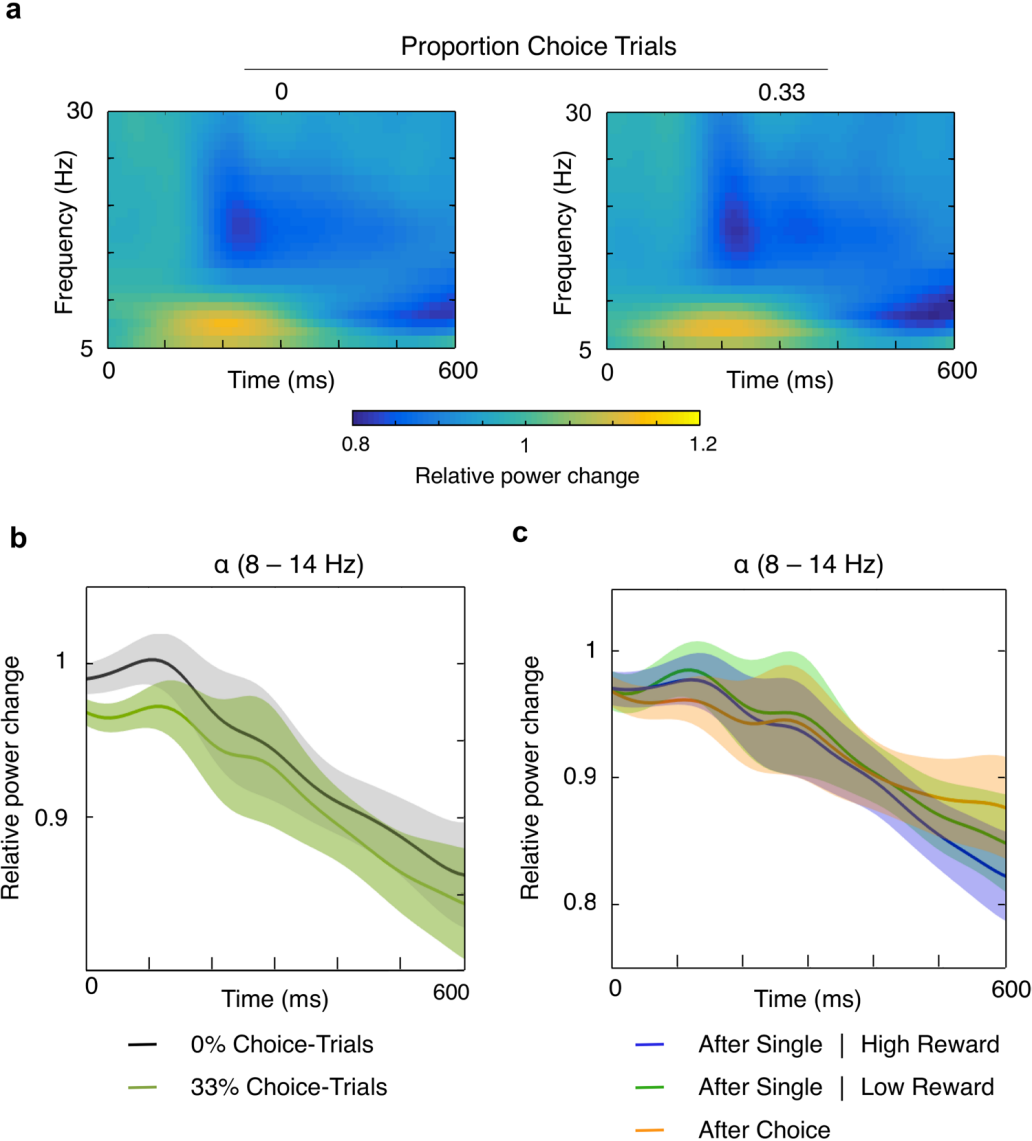
Electrophysiological results. (**a**) Time-frequency representations of the preparatory period in single-trials for the different choice-trial proportions (0 to the left and 0.33 to the right). Shown is the power change relative to a preceding baseline period at parieto-occipital electrode sites for frequencies of 5 to 30 Hz. A value of 1 indicates no change, values greater than 1 indicate a relative power increase, and values smaller than 1 indicate a relative decrease. (**b**) Relative power change in the alpha band (8–14 Hz) at parieto-occipital electrode sites across the preparatory period, shown separately for each proportion of choice-trials (0 in black, 0.33 in green). Shaded areas show the standard errors of the means. (**c**) Relative power change in the alpha band (8–14 Hz) at parieto-occipital electrode sites across the preparatory period, plotted separately for single-trials following either a single-trial with a high-reward target (blue line), a single-trial with a low-reward target (green line) or a choice-trial (orange line). Shaded areas show the standard errors of the means.

Figure 3a shows time-frequency representations of the preparatory period in the two different choice-trial conditions. Power estimates were computed relative to a 500 ms baseline period preceding the preparatory period, so that a value of 1 indicates no change, values greater than 1 indicate an increase, and values smaller than 1 indicate a decrease in alpha power. Changes in parieto-occipital alpha power in preparation for the upcoming target presentation were analyzed for an early (100–300 ms) and a late (300–500 ms) time window during the 600 ms preparatory period. Figure 3b shows the relative power change estimates across the preparatory period averaged across the alpha-band (8–14 Hz). As can be seen, alpha power decreased during the preparatory period. This was confirmed by a significant effect of time window (early time window: 0.96 ± 0.03; late time window: 0.91 ± 0.02; F_(1,22)_ = 12.63, p = 0.002, partial ƞ^2^ = 0.37). However, there was neither an effect of choice-trial proportion (F_(1,22)_ = 2.41, p = 0.135, partial ƞ^2^ = 0.10) nor an interaction of time window and choice-trial proportion (F_(1,22)_ = 0.66, p = 424, partial ƞ^2^ = 0.03). Thus, there was an overall decrease in alpha power in preparation for target processing, but this decrease was equivalent with and without choice-trials present.

We further examined intertrial effects in the 33% choice-trial condition, so as not to miss a reward-related preparation that was only initiated directly after a choice for a high-reward target had been made. Figure 3c shows the relative alpha power change across the preparatory period separately for single-trials following either a single-trial with a high-reward target, a single-trial with a low-reward target or a choice-trial. The general decrease in alpha power during the preparatory period was again confirmed by an effect of time window (F_(1,22)_ = 11.05, p = 0.003, partial ƞ^2^ = 0.33). But, as is quite apparent in Fig. 3b, the preceding trial type did not modulate overall alpha power (F_(2,44)_ = 0.11, p = 0.90, partial ƞ^2^ = 0.01) or the alpha power decrease (F_(2,44)_ = 0.60, p = 0.556, partial ƞ^2^ = 0.026).

## Discussion

In the present study, we investigated whether responses to single targets associated with different levels of reward are modulated by interleaved choices when reward is coupled to a nonspatial feature (i.e., color) as is the case when reward is coupled to spatial location^11,12^. Indeed, responses to single targets associated with a low reward were found to be delayed when choice-trials were present, magnifying the effect of reward on reaction times, while responses to high-reward single targets were not influenced by the manipulation of choice-trial proportion. The analysis of intertrial effects revealed that this selective slowing of responses to low-reward targets was particularly pronounced for single targets directly following a choice-trial, whereas responses to low-reward single-trials following a high-reward single-trial were not similarly delayed. This confirms that the increased reaction times to low-reward targets after a choice-trial were due to the previously made choice rather than due to a change in the color participants responded to from one trial to the next. Notably, the intertrial effects could not fully account for the response delay induced by the presence of choices (see also Wolf *et al*.^11^): Irrespective of the preceding trial type, responses to low-reward targets were slower with 33% choice-trials in the same block than without interleaved choice-trials. Overall, these findings mirror the pattern of results we obtained in our previous studies^11,12^, in which the level of reward was coupled to spatial location, and thus strongly suggest that choices modulate a reward-related bias in feature-based attention in a similar way as they do for spatial attention.

There is one aspect, in which the pattern of behavioral results differs from our previous findings. We failed to observe any effect of reward in blocks without choice-trials when reward was indicated spatially^11,12^, but in the present study, there was an effect of reward on reaction times to single targets even in the 0% choice-trial condition. However, it seems likely that this difference between studies with respect to reward effects without choice-trials is a quantitative but not a qualitative difference. In our previous experiments, there was always a consistent trend for slower performance for low-reward than for high-reward targets, and the effect observed in the present study, while larger and significant, was still rather small. That the effect of reward in the absence of choices was somewhat larger in the present study might be related to task-difficulty. The possibility to make choices and thereby maximize positive outcome is only one way by which reward can be assigned particular behavioral relevance. Another way is increased task-difficulty: With increasing task difficulty, the risk of making a mistake and losing reward altogether is higher. Therefore, biasing visual processing in a reward-related manner is a sensible strategy, which ensures that a mistake infers only a small cost (i.e., the loss of a low reward). The task in the present study was presumably slightly more difficult than the task we previously used^11,12^, because the target in single-trials was presented along with a distractor and had to be selected based on its color. That task difficulty was indeed higher with this paradigm is evident from overall longer reaction times and lower accuracy compared to our previous results. Correspondingly, the effect of reward on reaction times was slightly larger here than in our previous studies (16 ms as compared to 9 ms in Heuer *et al*.^12^). This is consistent with our finding that increased difficulty to make optimal choices, manipulated by varying the contrast of the targets, increases the delay in responses to low-reward targets^11^. The notion that a reward-related bias is only implemented when behavior can be optimized to maximize reward also reconciles our findings with the large body of research showing that reward affects visual processing in tasks that do not provide analogous choice opportunities (e.g., typical visual search tasks). These tasks are usually more difficult, for instance due to the presence of more distractors, so a reward-related bias ensures that mistakes will not be overly detrimental for the overall outcome^4,38–40^.

To examine whether the same oscillatory mechanism that we have previously identified for spatially indicated reward^12^ also supported the reward-related bias in feature-based attention observed in the present study, we additionally analyzed alpha-oscillations over parieto-occipital cortex during the preparatory period preceding target presentation. There was an overall decrease in alpha power during this period in all conditions, indicating that neural excitability in visual cortex was increased in order to facilitate processing of the upcoming target. However, even though the behavioral results clearly indicated that the feature associated with a low reward was effectively and more strongly suppressed when choices were present and especially so immediately following a choice-trial, this pattern was not reflected in posterior alpha power.

It might be tempting to assume that this lack of any reward-related modulation of posterior alpha oscillations shows that the suppression of low-value features was supported by a different neural mechanism than the suppression of low-value regions of space. This could be regarded as in line with previous findings. Of particular interest in this context is the study by Wildegger *et al*.^37^, who cued either the location, orientation, both or neither of an upcoming target stimulus and examined preparatory alpha modulations over visual cortex. While the anticipation of target location with spatial and combined spatial and feature cues was reflected in robust alpha lateralizations, preparing for a target feature (i.e., orientation) modulated neither lateralized nor global alpha power, even though the feature cues yielded clear performance benefits. The authors proposed that preparatory alpha modulations reflect a spatial gating mechanism that is involved in the gating of information processed by nonoverlapping sensory areas. This is for instance the case for different retinotopical locations^17,18^ but also for different feature dimensions^36^, which are processed in dedicated, separate areas. By contrast, alpha modulations would not operate at the level of specificity that is required when overlapping and interdigitating populations process the attended information^37^. This account accordingly predicts that no alpha modulations would be observed when feature values within the same dimension are attended, for example different orientations as in Wildegger *et al*.^37^ or different colors as in the present study. Along these lines, our results could be seen as extending empirical support for this idea put forward by Wildegger *et al*.^37^ to another feature dimension (i.e., color).

It is important to note, however, that there are plausible alternative explanations that could account for this null effect. For one, our measure might not have been sufficiently sensitive. We reasoned that the suppression of the low-reward feature would be mediated by increased alpha power (i.e., reduced excitability) in neural networks coding for that feature and that this would be reflected in overall higher alpha power. Possibly, our aggregate measure of electrophysiological activity of large populations of neurons in visual cortex recorded at the scalp surface was not sensitive enough to capture the changes in the oscillatory power of much smaller subpopulations. Moreover, the changes induced by the presence of choice-trials in the present study might even have been particularly subtle, seeing as only 33% of trials were choice-trials. The advantage of this design is that it controls for frequency effects: With a choice-trial-proportion of 33%, all conditions (choice-trials, low-reward single-trials and high-reward single-trials) have the same number of trials, which means that also all item types (low-reward target, high-reward target and distractor item) are presented equally often. But our previous work has shown that a higher proportion of choice-trials results in a larger response delay and presumably in a stronger underlying bias, so it is conceivable that a higher proportion of choice-trials might be required for oscillatory changes to be detected (see also Heuer *et al*.^11^ and Wolf *et al*.^12^ for discussions of the implications of different choice-trial proportions).

In summary, we have shown that the possibility to make choices modulates the effects of reward coupled to a non-spatial feature. Similar to what we have found for effects of reward coupled to spatial location^11,12^, responses to low-reward single targets were more delayed when choices between targets of different value were interleaved. Presumably, this is the result of an anticipatory bias in feature-based attention: Suppressing the feature value associated with a low reward in preparation of target presentation ensures that the more valuable target will be more readily selected when given the opportunity to make a choice. At a broader level, our findings support the notion that reward primarily affects performance when it is of immediate behavioral relevance, for instance due to the possibility to maximize positive outcome by making choices.

## Methods

### Participants

Twenty-six students of Philipps-Universität Marburg participated in the experiment. The data from three participants had to be excluded: Two because of technical problems that unsystematically distorted the EEG markers, and one because of excessive alpha activity. Analyses were performed on the data of the remaining twenty-three participants (18 female, five male; mean age 21 years, range 19–31 years). The experiment was conducted in accordance with the ethical standards laid down in the Declaration of Helsinki and approved by the Ethics Committee of the Faculty of Psychology. All participants provided informed written consent, were naive to the purpose of the experiment, and had normal or corrected-to-normal visual acuity and color vision. Visual acuity and color vision were tested with the OCULUS Binoptometer 3 (OCULUS Optikgeräte GmbH, Wetzlar, Germany).

### Apparatus and stimuli

The experiment was conducted in a dimly-lit and electrically shielded room. Participants were seated in a comfortable chair and were facing a monitor (22″, 1680 × 1050 px) at a viewing distance of 104 cm. Stimulus presentation and response collection were controlled by a Windows PC using E-Prime 2.0 software (Psychology Software Tools, Inc.). Participants responded by pressing buttons on the back of a gamepad (Microsoft SideWinder USB) with their left or right index finger. Three isoluminant colors were used as target and distractor colors: blue, green and yellow. All other stimuli were black and all stimuli were presented on a grey background. Target and distractor items as well as the small fixation cross shown during the preparatory period and during target presentation all had a size of 0.55° of visual angle. The large fixation crosses presented at the beginning of each trial and between trials, and the reward feedback presented at the end of each trial subtended 1.10°. Target and distractor items appeared 9.84° left or right from fixation.

### Procedure and Design

Figure 1 depicts the trial procedure. For the first 500–1000 ms of every trial, a fixation cross was shown. This presentation duration varied in randomly chosen steps of 100 ms. Until target and distractor onset, two placeholders (small fixation crosses) were presented at the upcoming target positions. The central fixation cross changed its size indicating the onset of the 600 ms preparatory period. Then, two circle-shaped items of different colors appeared, replacing the placeholders. For each participant, two out of three colors were defined as target colors. In the example illustrated in Fig. 1, green and blue are target colors, and the third color (yellow) served as distractor color. The color assignment was balanced across participants. In single-trials, one of the items was a target, as defined by its color, and participants had to indicate whether the target was presented to the left or right from fixation by pressing the spatially corresponding button (left or right) on a gamepad. In choice-trials, both items were targets, and participants could freely choose by pressing the left or the right button. The target was displayed until response or for a maximum of 700 ms. Participants received a reward for correct responses within this reaction time window. In each block of trials, one of the two target colors was assigned a low and the other one a high reward. In single-trials, correct responses were rewarded with either a low reward (+1 point) or a high reward (+9 points), depending on the target color. In choice-trials, the reward depended on the color of the chosen target. At the end of the experiment, reward points were converted into a monetary reward (35 Cents for 1000 points). Reward feedback (“+1”, “+9” or “+0”) at the end of each trial was presented for 700 ms. Inter-trial intervals varied randomly between 500 and 1000 ms in steps of 100 ms.

The experiment comprised 1728 trials in total. The two choice-trial proportions (0 vs. 0.33) were crossed with the assignment of reward to the two target colors (color 1 low, color 2 high vs. color 1 high, color 2 low) and varied blockwise (four blocks of 432 trials each). In 0% choice-trial blocks, half of all trials were low-reward single trials and the other half were high-reward single-trials. In 33% choice-trial blocks, one third of trials were low-reward single-trials, one third were high-reward single-trials, and the remaining third were choice-trials. Choice-trial proportion changed after each block and the reward assignment changed after two blocks (i.e., after the first half of the experiment). Within blocks, trial types (low-reward single-trial, high-reward single-trial, choice-trial) were chosen randomly. This design was disclosed to the participants. We balanced the order of the blocks across participants. Within each block, participants could take a short rest every 36 trials.

### Behavioral analyses

We excluded trials if the reaction time was more than 2.5 SD above the individual mean reaction time. This applied on average to 2.1% of all trials. The dependent variable of interest were reaction times in single-trials. Mean reaction times, including only trials with correct responses, were calculated separately for each proportion of choice-trials (0 vs. 0.33) and for low- and high-reward targets, and compared using a two-way repeated measures ANOVA. The same analysis was also computed for accuracy in percent to ensure that reaction times were not affected by a systematic trade-off between speed and accuracy. Moreover, we examined the percentage of high-reward choices in choice-trials as a manipulation check.

To examine intertrial effects, single-trials in the 33% choice-trial condition were sorted according to the preceding trial (choice-trial vs. low-reward single-trial vs. high-reward single-trial) and the reward associated with the target (low vs. high), and analyzed with a two-way repeated measures ANOVA.

### EEG recording and analyses

The EEG was recorded with 64 Ag/AgCl active electrodes (actiCAP, Brain Products, Munich, Germany) positioned according to the International 10–20 system. We recorded the horizontal (hEOG) and vertical electrooculogram (hEOG) as the voltage difference between electrodes positioned to the left and right of the eyes, and above and below. All electrodes were referenced to FCz and re-referenced offline to the average of all electrodes. Impedances were kept below 5 kΩ. The signal was recorded at a sampling rate of 1000 Hz with a high cutoff filter of 250 Hz and a low cutoff filter of 0.016 Hz.

Oscillatory activity in the preparatory period was analyzed in essentially the same way as in our previous study^12^ to facilitate comparison of the findings. However, in contrast to our previous study, we did not compute a lateralization index to investigate hemispheric differences in alpha power, because reward was no longer coupled to the visual hemifields. Instead, we examined parieto-occipital alpha power averaged across the hemispheres.

EEG preprocessing and analyses were performed in MATLAB (MathWorks) using the Fieldtrip toolbox^41^ and custom scripts. The continuous EEG was segmented into epochs of 2200 ms, starting 1000 ms before the onset of the preparatory period. We chose this comparatively long epoch to allow calculating wavelet coefficients for all frequencies and time points of interest, including the preparatory period and a preceding baseline from −500 to 0 ms. Trials that were incorrect, identified as reaction time outliers (>2.5 SD from individual mean reaction time), or that contained blinks (vEOG > 100 µV) or eye movements (hEOG > 70 µV) in the critical time window (−500 ms to 600 ms with respect to the onset of the preparatory period) were removed from the data. Segments were also excluded, when the absolute voltage in the channels of interest (O1/2, PO3/4 and PO7/8) exceeded 80 µV.

Time-frequency representations of the preparatory period in each trial were computed by convolving 5-cycle Morlet wavelets with the EEG segments for frequencies from 5 to 30 Hz with a resolution of 1 Hz. We applied this procedure in steps of 10 ms throughout the preparatory period and the preceding baseline of 500 ms for three electrode pairs over parieto-occipital cortex (O1/2, PO3/4 and PO7/8). Power estimates were baseline-corrected by dividing by the average power in the 500 ms preceding the onset of the preparatory period at each frequency. The resulting values thus reflect the change in power relative to the baseline period: a value of 1 indicates no change, values greater than 1 indicate a power increase, and values smaller than 1 indicate a power decrease. The relative power change estimates were averaged across electrodes and across frequency bins in the alpha range (8–14 Hz), separately for the 0% and 33% choice-trial condition. We excluded the first and last 100 ms of the preparatory period from the analysis so that it would not be affected by perceptual processing of the fixation cross change (i.e., the onset of the preparatory period) and the target. The remaining 400 ms were divided into an early (100–300 ms) and a late (300–500 ms) time window of analysis to ensure that more transient changes in alpha power would not be missed. These relative alpha power change estimates were submitted to a two-way repeated measures ANOVA with the factors proportion of choice-trials (0 vs. 0.33) and time window of analysis (early vs. late).

To examine intertrial effects, trials in the 33% choice-trial condition were further split according to the preceding trial type (choice-trial vs. low-reward single-trial vs. high-reward single-trial), and the resulting relative alpha power change estimates submitted to a two-way repeated measures ANOVA with the additional factor time window of analysis (early vs. late).

## Data Availability

The data are available at the following 10.5281/zenodo.1453309.
